# Bioinspired color-near infrared endoscopic imaging system for molecular guided cancer surgery

**DOI:** 10.1117/1.JBO.28.5.056002

**Published:** 2023-05-26

**Authors:** Mebin B. George, Benjamin Lew, Steven Blair, Zhongmin Zhu, Zuodong Liang, Indrajit Srivastava, Austin Chang, Hyungsoo Choi, Kyekyoon Kim, Shuming Nie, Sunil Singhal, Viktor Gruev

**Affiliations:** a University of Illinois at Urbana Champaign, Department of Electrical and Computer Engineering, Urbana, Illinois, United States; b University of Illinois at Urbana-Champaign, Department of Bioengineering, Urbana, Illinois, United States; c University of Pennsylvania, Perelman School of Medicine, Department of Thoracic Surgery, Philadelphia, Pennsylvania, United States; d University of Illinois at Urbana Champaign, Beckman Institute for Advanced Science and Technology, Urbana, Illinois, United States; e University of Illinois at Urbana-Champaign, Carle Illinois College of Medicine, Urbana, Illinois, United States

**Keywords:** endoscopy, multiple fluorophore imaging, fluorescence-guided surgery, bioinspired image sensor, near-infrared contrast agents

## Abstract

**Significance:**

Fluorescently guided minimally invasive surgery is improving patient outcomes and disease-free survival, but biomarker variability hinders complete tumor resection with single molecular probes. To overcome this, we developed a bioinspired endoscopic system that images multiple tumor-targeted probes, quantifies volumetric ratios in cancer models, and detects tumors in *ex vivo* samples.

**Aim:**

We present a new rigid endoscopic imaging system (EIS) that can capture color images while simultaneously resolving two near-infrared (NIR) probes.

**Approach:**

Our optimized EIS integrates a hexa-chromatic image sensor, a rigid endoscope optimized for NIR-color imaging, and a custom illumination fiber bundle.

**Results:**

Our optimized EIS achieves a 60% improvement in NIR spatial resolution when compared to a leading FDA-approved endoscope. Ratio-metric imaging of two tumor-targeted probes is demonstrated in vials and animal models of breast cancer. Clinical data gathered from fluorescently tagged lung cancer samples on the operating room’s back table demonstrate a high tumor-to-background ratio and consistency with the vial experiments.

**Conclusions:**

We investigate key engineering breakthroughs for the single-chip endoscopic system, which can capture and distinguish numerous tumor-targeting fluorophores. As the molecular imaging field shifts toward a multi-tumor targeted probe methodology, our imaging instrument can aid in assessing these concepts during surgical procedures.

## 1 Introduction

Early detection of solid tumors is of paramount importance for the successful treatment of various types of cancers.^1^^,^^2^ A favorable prognosis can be achieved when localized dysplastic lesions are surgically removed at the earliest onset of the disease. For example, the 5-year post-surgical survival rate for patients diagnosed with early-stage breast and lung cancer is >90% and 70% to 90%, respectively.^3^^,^^4^ In contrast, a drastic decline in survival rates has been reported for patients with advanced-stage cancer (29% for breast and 6% for lung). With recent developments in miniaturized high-resolution cameras and multimodal optics, endoscopic imaging using white-light illumination has provided a pathway toward minimally invasive surveillance and surgeries to facilitate early detection of cancer, faster patient recovery time, lower tissue trauma, and enhanced visualization of critical tissues and organs compared to open surgeries.^4^^–^^7^ However, white light endoscopy provides limited detection of flat or deeply seated cancerous lesions due to insufficient morphological differences compared to healthy tissue, which can lead to unfavorable clinical outcomes.^8^^–^^10^ For instance, patients with lung cancer undergoing white light endoscopic surgeries have a recurrence rate of 29% to 88%.^4^^,^^11^^,^^12^ Similar trends have been reported for other types of cancer, such as breast, esophageal, sinonasal squamous, and colorectal cancers.^13^^–^^16^

Near-infrared (NIR) molecular imaging can address these shortcomings by targeting tumor-specific biomarkers and highlighting the location and extent of primary as well as metastatic tumors. Due to the tissue’s low autofluorescence, absorption, and scattering in the NIR spectrum, high target-to-background ratios can be achieved for both superficial and deeply seated tumors.^17^^–^^20^ However, inter- and intra-tumor heterogeneity caused by mutation and metastasis of cancer cells limits the detection efficacy of single targeting molecular probes leading to variable patient outcome.^21^ Therefore, a cocktail of carefully selected and optimized NIR molecular probes targeting different tumor biomarkers can achieve higher tumor detection sensitivity and specificity compared to single tracers.^22^^–^^27^ The multi-tracer approach is the next frontier for molecular-guided cancer surgery because of the availability of a plethora of tumor-targeted probes that are either Food and Drug Administration (FDA) approved, such as pafolacianine for targeting folate receptors,^28^^,^^29^ or nearing approval from regulatory agencies around the world.^22^ However, most FDA-approved NIR imaging instruments, especially the ones focused on endoscopic surgeries, are optimized to detect fluorescence from a single NIR tumor-targeting probe and are not suitable for imaging cocktails of molecular probes. Imaging two NIR fluorophores requires the use of multiple imaging sensors leading to bulky endoscopic instruments with limited clinical translation.

Recently, our group reported the development and clinical evaluation of a single-chip hexa-chromatic imaging sensor.^30^ Our bioinspired imaging sensor (BIS), which is modeled after the visual system of the mantis shrimp, combines vertically stacked photodetectors with pixelated spectral filters to make three observations in the NIR spectrum while simultaneously capturing color images. This approach is radically different from current state-of-the-art multispectral imaging systems, which combine multiple cameras and complex optical elements, while still only imaging single molecular tracers. Imaging multiple tracers require additional cameras, which increases both size and cost of the device. BIS can image at least three NIR fluorophores between 700 and 1000 nm because of its unique pixel architecture. Furthermore, this new imaging approach enables the differentiation of fluorophores that could be excited using the same excitation source and has emission peaks that are 20 nm apart—a feature that is not possible in FDA-approved imaging instruments. Combining our multispectral imaging device with FDA-approved endoscopes yields suboptimal results in terms of spatial resolution and fluorophore limit of detection due to chromatic aberrations introduced in the endoscopes. Hence, a holistic design is needed to develop and clinically translate color-NIR endoscopic systems capable of imaging multiple NIR probes.

In this paper, we describe a novel rigid endoscopic imaging system (EIS) capable of simultaneously imaging and resolving two NIR fluorophores while concurrently capturing color (i.e., anatomical) images. The EIS is composed of a rigid endoscope that is optimized for NIR-color imaging, custom illumination fiber bundle, and BIS. We present data from the benchtop to the operating room. Initially, we investigate the enhanced optoelectronic performance of EIS and compare it with an FDA-approved NIR endoscope from Karl Storz (KS). Next, we demonstrate the system’s imaging capability to discriminate two small molecule NIR tumor-targeted probes, first in vials and then in a murine metastatic breast cancer model. Finally, we provide clinical data from patients diagnosed with non-small cell lung cancer. NIR fluorescence from the cathepsin-activated probe in the tumor site is imaged *ex vivo* in the operating room with EIS. These back-table experiments in the operating room demonstrate the feasibility of using our imaging instrument for the detection of tumors tagged with NIR fluorescent probes in patients with lung cancer. Discussion is presented at the end of the paper.

## 2 Materials and Methods

### 2.1 EIS Design

The EIS consists of a 4 or 10 mm rigid endoscope (OMEC Medical, San Jose, California, United States), multiple custom bifurcated fiber cables (Sunoptic Technologies LLC, Jacksonville, Florida, United States) and BIS, which is coupled to the endoscope at the proximal end. The custom bifurcated fiber cables are used to couple laser light excitation from a 665 nm laser light source (BWF2-665, B&W TEK, Plainsboro, New Jersey, United States), a 785 nm laser (R0785MU6000M4S, Innovative Photonic Solutions, Plainsboro, New Jersey, United States) and broadband light-emitting diode (LED) visible light (UHP-T- WDS-DI, Prizmatix, Givat -Shmuel, Israel) to the endoscope’s light post. BIS is coupled to the scope using a C-mount camera coupler. Two notch filters are placed in the camera housing to suppress excitation source light at 665 nm (NF03-658E, Semrock) and 785 nm (NF03-785E, Semrock). The image sensor is housed in a custom-built camera housing which was optimized for real-time data acquisition while minimizing external electromagnetic interference. All custom-printed circuit boards and camera housings are designed using Altium and AutoCAD software, respectively, and fabricated by PCBWay (Shenzhen, China). A data acquisition board inside the camera housing (OpalKelly XEM 7310, Seattle, Washington, United States) receives data from the image sensor using a low voltage differential signal bus and transfers the image data via a USB 3.0 interface to an external computer. The camera’s firmware program is written in Verilog language. Data acquisition, image processing, and display are developed in Python, enabling real-time data acquisition and display on a Mac Studio (M1 Ultra processor, 128 GB unified memory, 8 TB solid state hard drive, Apple, Cupertino, California, United States). Video data files are saved in h5 data format, which includes video data and various metadata information. The same setup (illumination sources, bioinspired camera, and computer) was used to evaluate the KS endoscope 1.9 mm (64301AA, Karl-Storz, Tuttlingen, Germany).

### 2.2 Bioconjugation of IRDye 680RD with Cyclic RGD Peptides

IRDye 680RD was conjugated with integrin-specific cyclic arginylglycylaspartic acid peptide (cRGDfK, Vivitide, Gardner, Massachusetts, United States) as previously reported.^31^^,^^32^ Briefly, IRDye 680RD-NHS (0.5 mg, LI-COR Bioscience, Lincoln, Nebraska, United States) was dissolved in 0.5 ml phosphate buffered saline (PBS) at pH 7.2 under gentle stirring. Subsequently, the cRGDfK dissolved in 0.5 ml PBS was added to the solution and stirred for 2 h at room temperature. The cRGDfK-conjugated IRDye 680RD (IR680-integrin) was then purified by dialysis. The concentration of the dye after the conjugation was determined by measuring the absorbance at 680 nm using a ultraviolet-visible (UV-Vis) spectrophotometer (Genesys 10s UV-Vis Spectrophotometer, Thermo Scientific, Waltham, Massachusetts, United States).

### 2.3 Bioconjugation of IRDye 800CW with epidermal growth factor-specific Affibodies

Epidermal growth factor (EGF)-specific affibody ($Z_{\mathrm{EGFR}}$, 0.25 mg, Affibody AB, Solna, Sweden) was initially reduced with tris (2-carboxyethyl) phosphine (TCEP, Sigma-Aldrich, St. Louis, Missouri, United States) in 0.5 ml PBS for 30 min at room temperature, as previously reported.^33^ Afterward, IRDye 800CW-maleimide (0.5 mg, LICOR Bioscience, Lincoln, Nebraska, United States) dissolved in 0.5 ml PBS was added to the solution and stirred for 2 h at 40°C. The $Z_{\mathrm{EGFR}}$-conjugated IRDye 800 (IR800-EGF) was subsequently purified by dialysis. The concentration of the dye after the conjugation was determined by measuring the absorbance at 775 nm using a UV-Vis spectrophotometer.

### 2.4 Spatial Resolution Optical Evaluation

The spatial resolution of EIS and KS endoscope coupled with BIS was evaluated using a United States Air Force (USAF) 1951 resolution target (Edmund Optics, Barrington, New Jersey, United States) and a depth of field target (DOF 5-15, Edmund Optics, Barrington, New Jersey, United States). The USAF resolution chart was back-illuminated using an integrating sphere (IS200-4, Thorlabs, Newton, New Jersey, United States) coupled with two light sources: a visible LED and a 785 nm laser light source. The integrating sphere ensured uniform illumination of the target. The endoscope was initially focused under white light illumination using color images. The working distance for the endoscopes was adjusted depending on the groups targeted on the USAF chart (10 mm for groups 1 and 2, and 0.2 mm for groups 4 and 5). Subsequently, the white light was replaced with NIR illumination without readjusting the focus to acquire NIR images. The vertical elements 1 to 6 in group 5 within the USAF target were then selected to determine the spatial resolution in terms of line pairs per millimeter (lp/mm).

The DOF target was placed at 45 deg relative to the endoscope’s field of view (FOV), targeting the horizontal bars with 15 lp/mm. The white light illumination was utilized to focus the bars at a specific depth within the scope’s FOV. Next, the white light was replaced with 785 nm illumination to acquire NIR images without readjusting the focus. For analysis, the intensities of a row of pixels within the endoscope’s FOV were plotted at different depths. The same set of pixels was used for the visible and NIR images. The results from the two endoscopes, under the same testing conditions, were compared to assess each scope’s capabilities to address focal plane shift between visible and NIR images. Data were recorded using our custom software and analyzed using MATLAB scripts.

### 2.5 Fluorescence Sensitivity

The sensitivity of the system was evaluated by computing the mean fluorescence intensity (MFI) of each probe by averaging the digital value of all pixels within a selected region of interest (ROI). IR680-integrin, IR800-EGF, and indocyanine green (ICG) were prepared in a serial dilution (0.1 to 1  μM) in 2.0 ml vials in triplicate. PBS was used as a negative control. The limit of detection was defined as the concentration whereby the MFI of the probe exceeded the MFI of the negative control with a 95% confidence interval (two standard deviations of the mean). Fluorescence images were acquired using 665 nm laser excitation for IR680-integrin and 785 nm laser excitation for IR800-EGF and ICG at a 200 ms exposure setting [5 frames per second (FPS)]. The total output power of the 665 nm and 785 nm laser was 50 and $150\;\mathrm{mW}/{\mathrm{cm}}^{2}$, respectively, with the vial positioned 20 mm away from the distal end of the endoscope.

### 2.6 Spectral Resolution

A computer-controlled monochromator (Acton SP2150, Princeton Instruments, Trenton, New Jersey, United States) was used to evaluate the spectral sensitivity of our system. The monochromator was programmed to generate a sequence of spectral bands between 680 and 820 nm in 20 nm steps within the system’s FOV, and the data were recorded at each step for analysis. The hue-saturation response of each detected band was plotted with 68% and 95% tolerance ellipses.

### 2.7 Volumetric Analysis

IR680-integrin and IR800-EGF were mixed in vials in six different volumetric ratios (ranging from 10:0 to 0:10, 2.0 ml in PBS, n=3) and excited simultaneously with 665 and 785 nm laser illumination. All vials were imaged using EIS. The hue-saturation response of the detected fluorescence from each mixture was plotted with 68% and 95% tolerance ellipses.

### 2.8 Animal Study

Nine female immunodeficient mice (J:NU, 2 months old, average weight 25 g; The Jackson Laboratory, Bar Harbor, Maine, United States) were used for *in vivo* tumor imaging. Each mouse was inoculated with 4T1 cells ($1\times 10^{6}\text{ cells}$ per injection) orthotopically into one of the mammary fat pads. Once the tumor grew to about $1\;{\mathrm{cm}}^{3}$ in size, the nine mice were divided into three equal groups (i.e., three mice per group) and administered with 100  μl of either IR680-integrin, IR800-EGF, or an equimolar mixture of the two probes (100  μM). After 6 h post-administration, the mice were anesthetized with 1.5% to 2.0% isoflurane on a heated pad and imaged using EIS with the two light sources (665 and 785 nm) at 200 ms exposure time (5 FPS). ROI was selected around the tumor and adjacent healthy tissue to compute both the tumor-to-background ratio (TBR) and hue-saturation response for probe discrimination. The TBR was determined as the ratio of the fluorescence intensity of tumor ROI and fluorescence intensity of adjacent healthy tissue ROI. All animal experiments were performed under protocols approved by the University of Illinois Institutional Animal Care and Use Committee.

### 2.9 Ribonucleic Acid Sequencing Analysis

Expression levels of integrin (β3) receptors and EGF receptors (EGFRs) in 4T1 cells were assessed by transcript level expression analysis. Initially, ribonucleic acid-seq reads were downloaded from the Gene Expression Omnibus (GEO) database and processed for quality and read length filters using Trimmomatic (v0.38). They were then aligned to either the mouse genome (mm10) or human genome (hg38) using STAR (v2.7.3). Gene expression levels were quantified across samples using the cuffnorm function in Cufflinks (v2.2.1) and were compared in transcripts per million.^34^^–^^37^ The mean and standard deviation of each gene expression were obtained by averaging 10,000 readings.

### 2.10 Back-table Imaging of Human Lung Cancer Nodules

We used EIS to image lung cancer nodules from patients diagnosed with non-small cell lung cancer at the hospital of the University of Pennsylvania. All required authorizations were approved by the University of Pennsylvania Institutional Review Board. At 24 h prior to the surgery, 0.32  mg/kg of VGT-309 (Vergent Bioscience, Minneapolis, Minnesota, United States) was administered intravenously to 6 patients. VGT-309 is a cathepsin-binding ICG agent that could selectively activate fluorescence signals in the tumor microenvironment where there is an upregulation of cathepsin.^38^ The surgeon used a NIR intraoperative imaging system (VisonSense, Medtronic, Philadelphia, Pennsylvania, United States) to identify the location of the malignant nodules tagged by VGT-309 and performed wedge-resection containing the tumor and healthy tissue. Subsequently, EIS with a 785 nm laser was used to image each nodule *ex vivo* on the back table at 200 ms exposure time (5 FPS). Afterward, the nodules were histologically analyzed to confirm their malignancy. The fluorescence intensity of the sectioned specimens was measured using a NIR fluorescence scanner (Odyssey CLx imager, Li-Cor, Lincoln, Nebraska, United States).

The captured images were processed to show the contrast between the tumor and its margins by plotting the distribution of the corresponding fluorescence intensities of the two regions. ROI was selected around the tumor and the healthy tissues to evaluate the TBR. The hue-saturation response of the fluorescence from the tumor was evaluated and compared with that of an ICG solution prepared in a vial (10  μM in PBS)

### 2.11 Statistical Analysis

Data prepared in triplicate were expressed as mean values ± SD. One-way analysis of variance with Tukey post-hoc was performed in Origin Pro software for statistical analysis. Differences between samples were denoted as significant at p<0.05.

## 3 Results

### 3.1 Endoscopic Imaging System

**Fig. 1:**
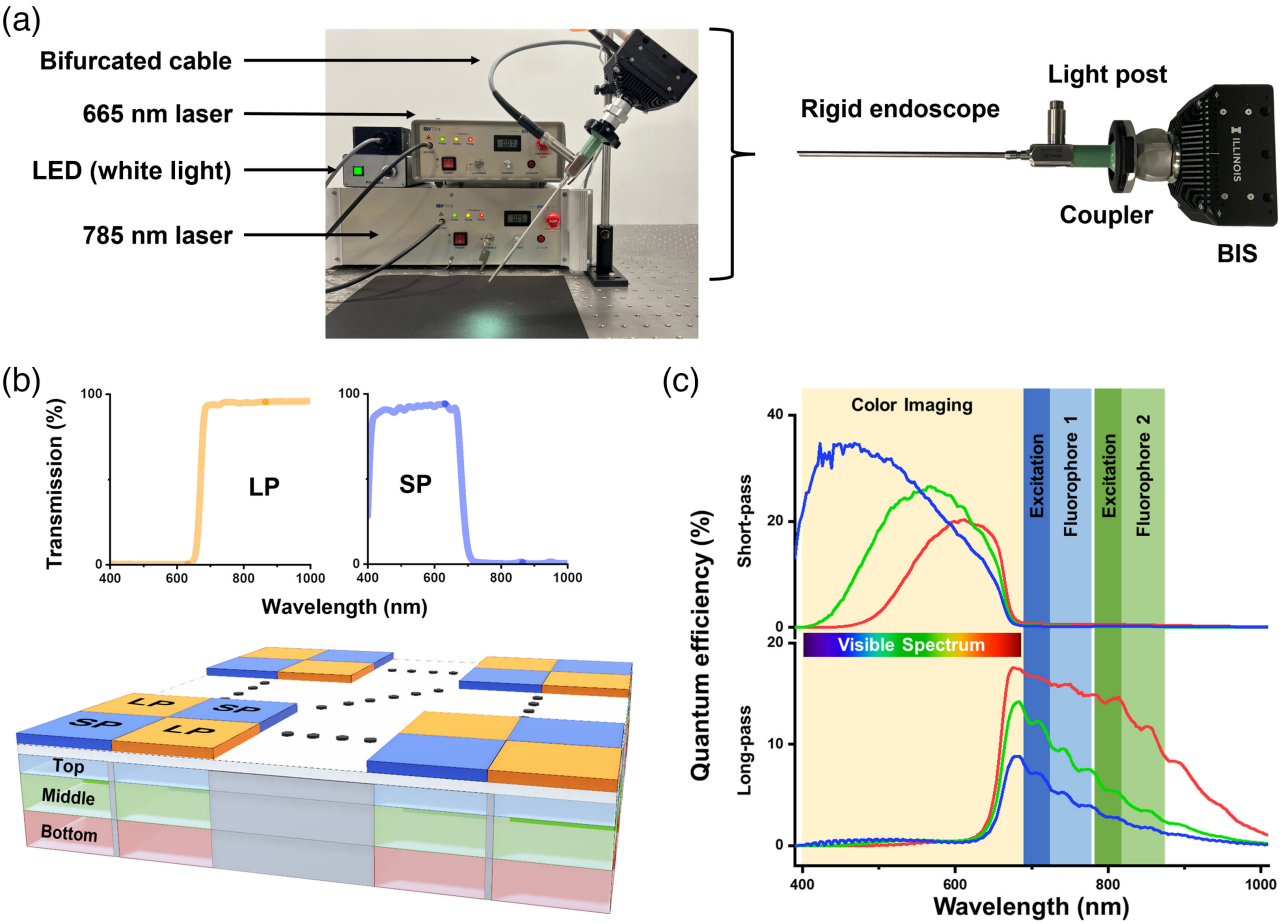
A snapshot of EIS for imaging multiple tumor-targeted NIR probes. (a) The system is comprised of a custom-designed rigid endoscope, fiber optical illumination bundle, 665 nm laser, 785 nm laser, and BIS.^30^ (b) The imaging sensor combines an array of vertically stacked photodiodes and pixelated spectral filters to sense six different spectral bands. (c) The quantum efficiency for the top, middle, and bottom photodiode under the LP and SP pixelated spectral filters.

Our EIS comprises a custom-designed rigid endoscope, fiber optical illumination bundle, and BIS [Fig. 1(a)]. The custom endoscope contains several cylindrical lenses with multiple optical coatings to minimize both optical losses in the NIR spectrum and chromatic aberrations between 400 and 900 nm wavelength range. This design ensures that both visible and NIR images are focused on the same imaging plane. The endoscope is fabricated by OMEC Medical System. In this study, three light sources (white LED, 665 nm laser, and 785 nm laser) were coupled into the light post of the endoscope using multiple custom-built bifurcated fiber cables. Two notch filters with optical density >6 at 665 and 785 nm were placed in the camera housing to suppress the laser excitation light.

Figure 1(b) displays the schematic representation of the pixel array of BIS, which consists of vertically stacked photodiodes and pixelated spectral filters.^30^^,^^39^ The pixelated spectral filters are arranged in a checkerboard pattern and alternate between short-pass (SP), which only transmits visible spectrum photons, and long-pass (LP), which only passes NIR photons. The out-of-band rejection ratio (i.e., optical density) of the pixelated filters is ∼4, which is about 2 orders of magnitude higher than commercial NIR-color sensors.^30^ The imaging device is fabricated in silicon, which has a wavelength-dependent absorption coefficient. Since the image sensor’s absorption coefficient is much higher in the blue spectrum compared to red and NIR spectra, lower wavelength photons are absorbed dominantly in the top photodiode compared to the middle and bottom photodiode. Hence, the top (bottom) photodiodes have preferential registration of the blue (red) spectrum for the visible pixels. For the NIR pixels, the bottom photodiode exhibits the highest quantum efficiency due to the high penetration depth of NIR photons in silicon detectors. Even though the quantum efficiency in the NIR spectrum decreases rapidly for the top and middle photodetectors, a total of three distinct measurements are achieved across this spectrum. The quantum efficiency for the three visible and three NIR photodiodes is shown in Fig. 1(c), indicating simultaneous imaging capabilities of both color and multiple NIR channels using a single-chip imaging device.

### 3.2 Optical Characterization

**Fig. 2:**
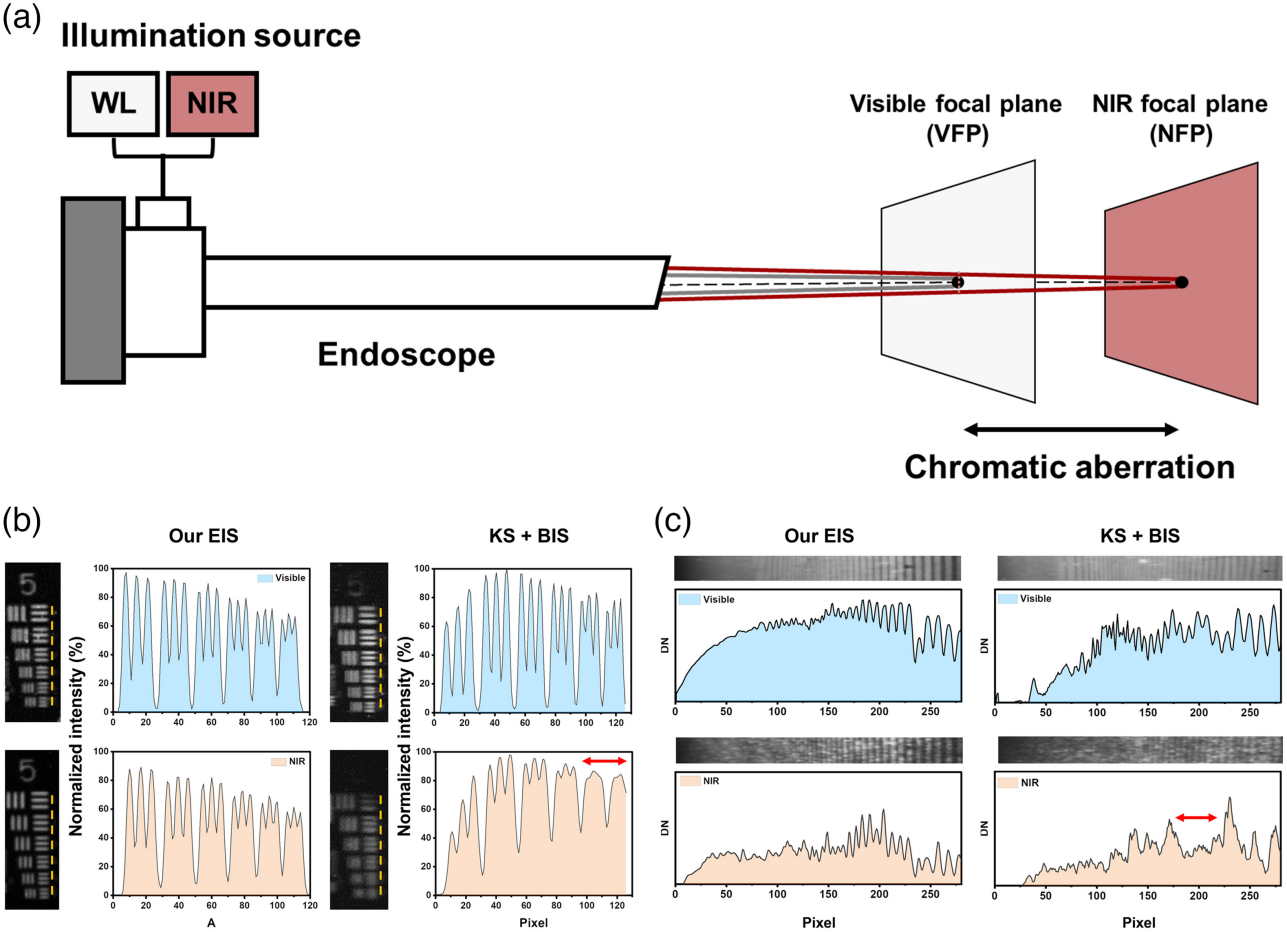
Spatial resolution of EIS and KS + BIS under white light and NIR illumination. (a) Chromatic aberrations in endoscopes lead to different imaging planes when illuminated with visible or NIR light (b) visible (top) and NIR images (bottom) of the USAF resolution chart. The resolution of the six elements in group 5 (yellow dotted line) was analyzed and plotted as pixel position versus intensity. (c) Images of an ROI in the slanted depth of focus target under visible and NIR illumination. The corresponding line profiles are plotted below. Red arrows indicate regions where the images in the NIR spectrum are aliased (i.e., blurred).

We performed a series of optoelectronics tests using EIS and compared the results with an FDA-approved endoscope from KS (FOV of 4.5 cm measured from a working distance of 2 cm from the object plane). First, we evaluated the impact of chromatic aberrations on the spatial resolution of EIS (FOV of 4 cm measured from a 2 cm working distance) and KS-NIR endoscope coupled with BIS (KS + BIS). Chromatic aberrations are typically introduced by the individual cylindrical lenses in the endoscope and can cause visible and NIR images to be focused on different imaging planes [Fig. 2(a)]. Consequently, if the endoscope is focused on features obtained with white light illumination (i.e., based on color images), the NIR images can be out of focus leading to a reduction in both spatial resolution and NIR sensitivity.

We imaged a USAF resolution chart using both endoscopes under separate white light and NIR illumination. Initially, the USAF target was illuminated with white light and the focus of the endoscope was adjusted based on the color images using micromanipulators. Next, the white light was turned off, and a 785 nm laser light was used to illuminate the target. The focus of the endoscope remained undisturbed between white light and NIR illumination. Figure 2(b) shows the intensity profile for both color and NIR images across the horizontal lines in group 5. From this figure, a good correspondence was observed between the NIR and color images captured by EIS when imaging high-frequency horizontal elements. The intensity contrast was reduced but differentiable even when high-frequency elements in group 5 were imaged. On the contrary, KS + BIS exhibited a noticeably lower spatial resolution in the NIR spectrum compared to the visible spectrum. In this endoscopic system, even though the color image remained in focus for elements with high spatial frequency, the NIR image was aliased (i.e., blurred) and could not differentiate high-frequency elements. The intensity profile indicates that EIS was able to resolve elements that were 9  μm apart (i.e., 57  lp/mm) for both color and NIR images. In contrast, KS + BIS demonstrated a spatial resolution of 9  μm under white light illumination but only 15  μm resolution under NIR illumination. This indicates that EIS has a 60% higher spatial resolution in the NIR spectrum when simultaneously imaging color images compared to KS + BIS.

Next, we imaged a depth-of-focus calibration target to emulate an endoscopic imaging scenario where targets with different spatial frequencies can appear simultaneously at different distances from the tip of the endoscope. Figure 2(c) shows visible images, NIR images, and intensity cross profiles for both imaging modalities. It can be observed that the color image registered by both endoscopes can resolve high spatial frequency patterns at different depths. However, the NIR image captured by KS + BIS was severely aliased, making it challenging to differentiate high spatial frequencies at increasing distances from the tip of the scope. In contrast, EIS was able to spatially resolve these variable frequency targets at different distances due to its higher spatial resolution in the NIR spectrum.

### 3.3 Limit of Detection for Small Molecule Tumor Targeted Probes

**Fig. 3:**
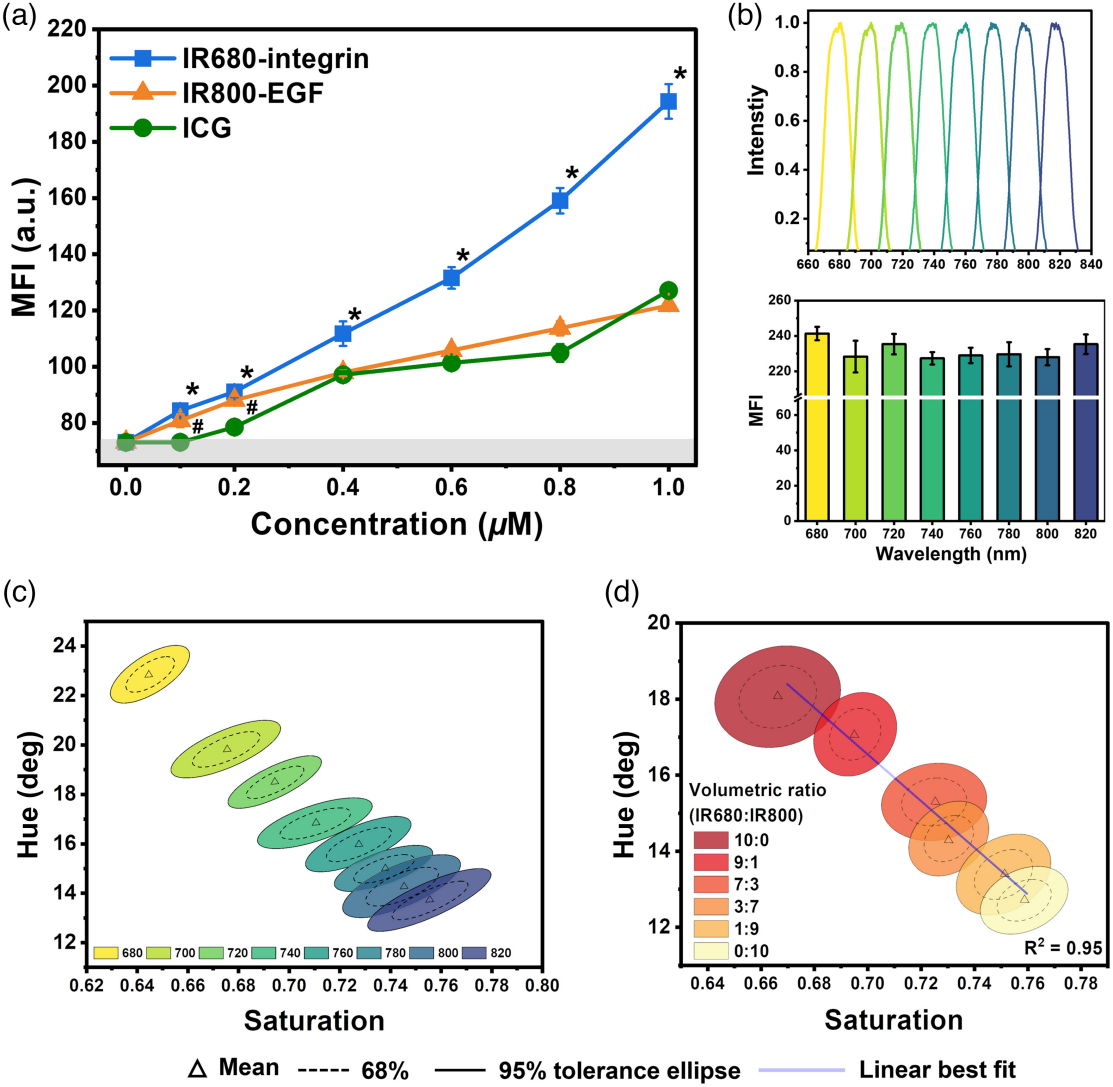
Limit of detection and spectral discrimination of EIS. (a) The sensitivity of EIS was analyzed using IR680-integrin, IR800-EGF, and ICG at varying concentrations (n=3). The gray area represents the background signal from a vial with PBS (95% confidence interval). The characters * and # denote the significance difference in MFI of IR680-integrin and IR800-EGF, respectively, with ICG (p<0.05). (b) The EIS was illuminated with eight different narrow-band lights generated from a monochromator. The MFI of these targets had no statistically significant difference (n=3, p<0.05). (c) EIS can spectrally discriminate these eight narrow-band targets despite their similar intensity profiles. The spectral response of EIS is represented by converting the raw intensity data from the three vertically stacked photodiodes into hue-saturation values. (d) Spectral discrimination capabilities of EIS when imaging different volumetric ratios of IR680-integrin and IR800-EGF. Each cluster represents the mean distribution with 68 % and 95% tolerance ellipses. Linear regression was applied to generate a best fit line within the clusters.

Small molecule-based tumor-targeted probes, such as peptide- or affibody-targeted probes, have fast renal clearance and enable high tumor-to-background imaging within a few hours post-administration.^40^^–^^42^ This is critical for same-day administration of the tumor-targeted probes followed by surgical procedures, which can reduce healthcare costs and avoid hospital infections by minimizing hospital stays. Therefore, we evaluated the limit of detection for two small molecule-based tumor-targeted probes: (1) IR680-integrin and (2) IR800-EGF. The limit of detection for ICG was also evaluated since it is routinely used for sentinel lymph node mapping and tumor demarcation for various cancer surgeries. Several different probe concentrations ranging from 0.1 to 1  μM were prepared in vials, excited with either 665 or 785 nm laser and imaged with EIS. Figure 3(a) displays the MFI recorded with EIS for the three probes, each exhibiting a linear increase from 0.1 to 1  μM. The highest rate of increase was observed with IR680-integrin, which exhibited a significantly higher MFI at each concentration compared to that of ICG due to the higher quantum yield of IRDye 680RD compared to ICG along with the sensor’s higher quantum efficiency at 700 nm compared to 800 nm wavelength. IR800-EGF and ICG exhibited a similar level of MFI at concentrations >0.2  μM. However, below 0.2  μM, the MFI of ICG was significantly lower than that of IR800-EGF. Overall, the limit of detection of EIS was evaluated to be 0.1  μM for all probes with 95% confidence above the background (i.e., PBS, 73.09±0.09).

### 3.4 Narrowband Spectral Sensitivity in the NIR Spectrum

One of the unique features of EIS is the ability to detect and differentiate multiple NIR spectral bands, which has not been featured in FDA-approved EISs. The image sensor enables NIR spectral imaging because it makes three observations in the NIR spectrum via vertically stacked photodetectors, each with distinct quantum efficiencies. Although this system can differentiate three different NIR fluorophores, we can also image a multitude of NIR fluorophores with distinct spectra. The analogy is similar to color imaging where three spectral photodetectors (namely red, green, and blue) can record a scene and differentiate thousands of different colors.

To demonstrate the NIR multispectral imaging capabilities of EIS, we first imaged 8 different narrow-band illuminations between 680 to 820 nm in increments of 20 nm and with a full-width half maximum of 17 nm [Fig. 3(b)]. The intensity for each illumination was matched (231.8±5.0) by adjusting the exposure setting of the image sensor. In other words, the eight spectral bands cannot be differentiated based on the intensity recorded by EIS or any other FDA-approved NIR endoscopic system. Since the NIR pixels make three observations, each spectral target produced a different intensity response in the three vertically stacked photodiodes. The raw intensity response from the three photodiodes was transformed to hue-saturation-value space, where the spectral content of the targets is represented by the hue and saturation components. Figure 3(c) depicts the mean confidence intervals in the hue-saturation space for all eight spectral bands. The spectral targets between 680 and 760 nm are spectrally separable with at least a 95% confidence interval or better. The spectral discrimination between 780 and 820 nm decreased due to the lower quantum efficiency for the top photodetector in the NIR spectrum.

### 3.5 Volumetric study for Multiple Fluorophore Differentiation

We evaluated the spectral differentiation capabilities of EIS by imaging various volumetric ratios of two small molecule-based tumor-targeted probes in vials: IR680-integrin and IR800-EGF. All vials were excited simultaneously with 665 and 785 nm lasers and the NIR fluorescence emission was measured with EIS. The three intensity measurements in the NIR spectrum from the vertically stacked photodiodes in the image sensor were converted to the hue-saturation space and displayed in Fig. 3(d). The higher uncertainty in the hue-saturation response from the probe mixtures compared to the narrow-band illumination experiments was due to the wider emission spectra of the molecular probes. Furthermore, similar to the monochromatic experiments, volumetric ratios where the emission spectrum was dominated by IR680-integrin had better differentiation than those where IR800-EGF was dominant. This was due to the lower quantum efficiency of the top photodiode at wavelengths above 800 nm, rendering lower signal-to-noise ratio signals obtained by the top photodiodes in this wavelength spectrum.

### 3.6 Dual Probe Imaging in Murine Breast Cancer Model

**Fig. 4:**
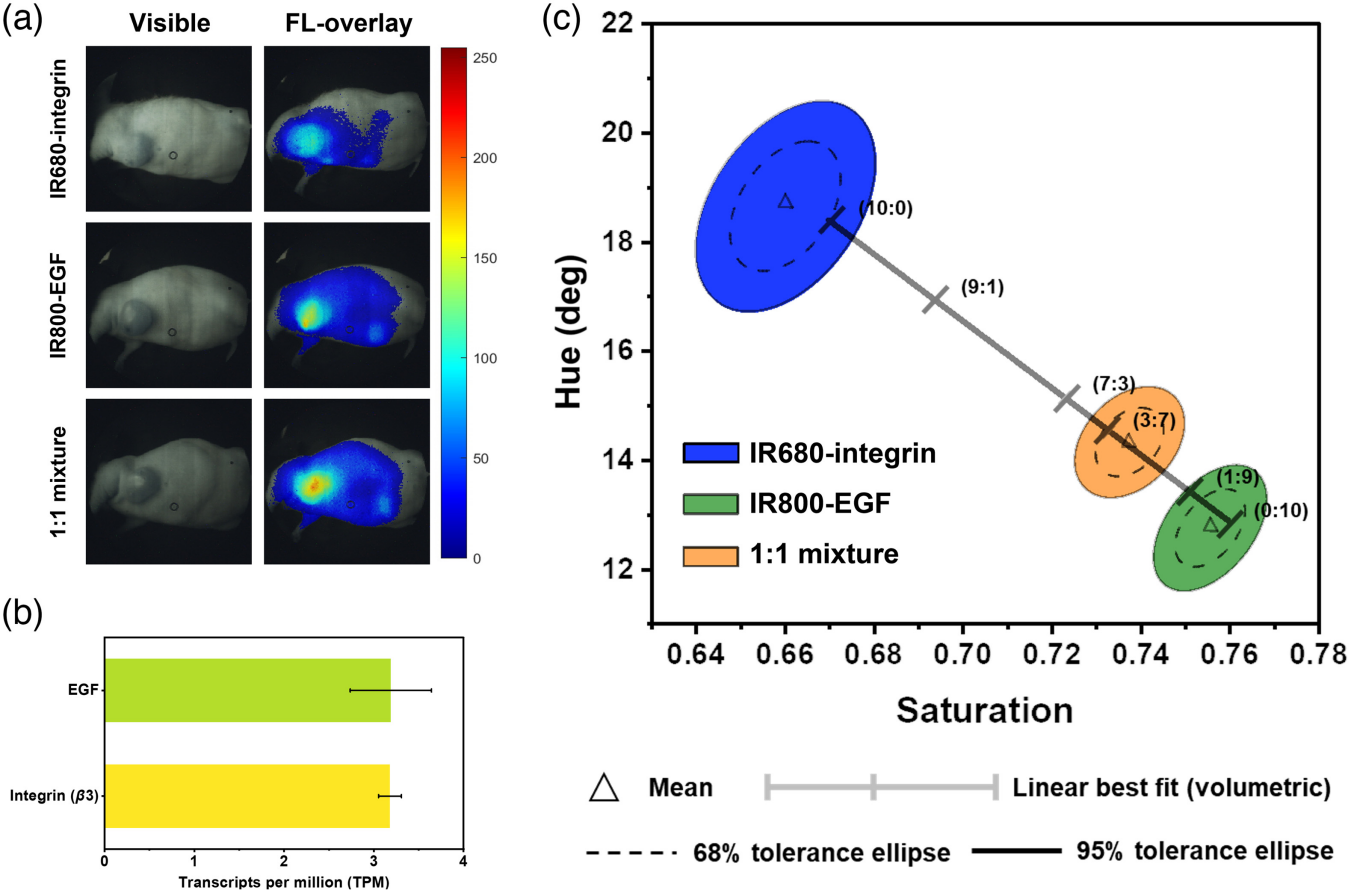
*In vivo* validation of EIS. (a) Visible and fluorescence-overlayed images of 4T1 tumor-bearing mice at 6 h post-administration of IR680-integrin, IR800-EGF, or 1:1 mixture of the two probes. (b) RNA transcripts of 4T1 tumors showing the expression level of EGFR and integrin receptor β3. Error bars represent the deviation of the samples from the mean. (c) Spectral discrimination of the two probes *in vivo* using EIS. The ratio of the mixed probes at the tumors was estimated by applying the best fit line from the vial study. Each tick represents the mean hue-saturation response of corresponding volumetric ratios of the two fluorophores.

Next, we evaluated the multispectral imaging capability of EIS by simultaneously imaging two tumor-targeted probes in a murine model of breast cancer. For these experiments, we used three groups of 4T1 tumor-bearing female nude mice. We administered either IR680-integrin or IR800-EGF probe in the first two groups and a mixture of both dyes with an equal molar concentration in the third group. At 6 h post-administration, all mice exhibited strong fluorescence from the tumor regions with minimal background fluorescence [Fig. 4(a)]. The first two groups of mice exhibited TBRs of 3.6±0.17 (IR680-integrin) and 3.3±0.07 (IR800-EGF), and the third group exhibited a TBR of 4.5±0.12. The higher TBR in the third group compared to the first two groups is due to the accumulation of both probes in the tumors, which leads to higher confidence in tumor detection.

We compared the fluorescence spectra from the tumors in three groups of mice by plotting the hue-saturation response recorded with the EIS. Although the fluorescent intensities from IR680-integrin and IR800-EGF do not have statistically significant differences in the first two groups of mice, both probes could be differentiated with 100% confidence using the hue-saturation spectral signatures [Fig. 4(c)]. Furthermore, the spectral signature from the third group of mice, where both probes were present in the tumors, had a distinct hue-saturation signature compared to the fluorescence from the first two groups of mice. The fluorescence from the three groups of mice exhibited a similar hue-saturation response compared to the vial study experiments, suggesting that the third group had 30% contribution from IR680-integrin and 70% from IR800-EGF [Fig. 3(d)]. An RNA transcript analysis of 4T1 tumor cells indicated similar expression levels for both EGFRs and integrin (β3) receptors (p>0.05), which are the target receptors for IR800-EGF and IR680-integrin, respectively [Fig. 4(b)]. Hence, we initially expected the *in vivo* results to exhibit the same level of fluorescence from the two probes at the tumor sites. However, the hue-saturation analysis indicated more dominant fluorescence from IR800-EGF, which could be attributed to many physiological factors including tumor heterogeneity, passive accumulation of the probes, light absorption, and scattering from the skin of the mouse.

### 3.7 Back-table Imaging of Human Lung Cancer Nodules

**Fig. 5:**
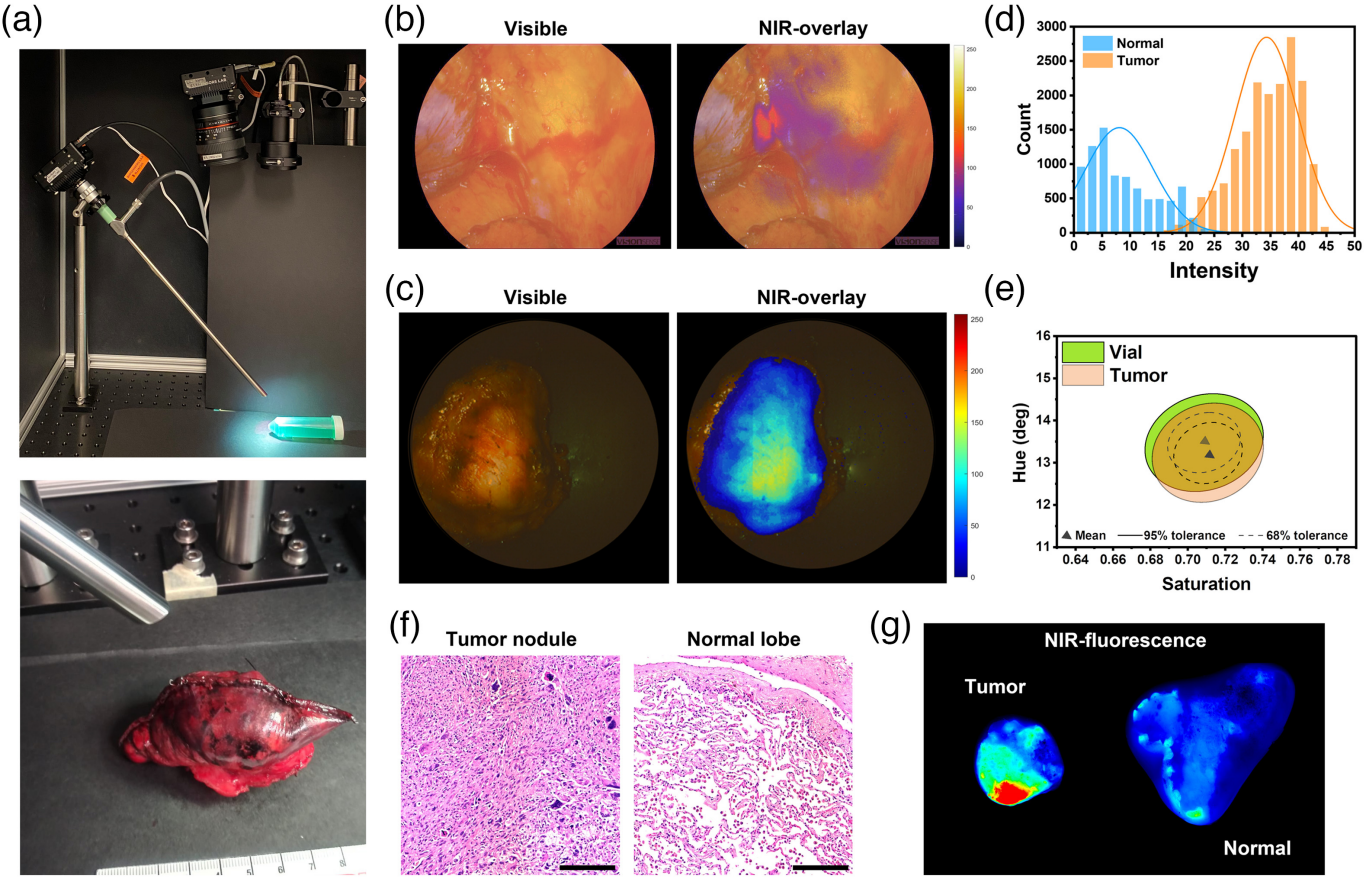
Clinical validation of EIS by imaging fluorescently labeled lung cancer nodules on a back table in the operating room. (a) Experimental setup of EIS for the back-table imaging of the resected lung nodules and lobes. (b) Visible and fluorescent images of lung cancer nodules acquired during the operation with NIR Intraoperative imaging system and (c) back-table imaging with EIS immediately after resection. (d) A histogram showing the distribution of fluorescence intensity of the regions of interest marked at the malignant nodules and normal tissue. (e) Hue-saturation response of fluorescence signal detected in the tumor compared to the signal in a vial. (f) Representative H&E images of a malignant nodule and normal tissue. Scale bar: 200  μm. (g) Fluorescence images of the resected tumor nodule and adjacent normal lobe acquired by a NIR scanner.

Patients diagnosed with non-small lung cancer and undergoing surgical treatment were recruited for an IRB-approved study at the hospital of the University of Pennsylvania. The patients were intravenously administered 0.32  mg/kg of VGT-039 24 h prior to surgery. During the operation, the surgeon performed a wedge resection to remove cancerous nodules that were fluorescently identified using a NIR intraoperative imaging system. Immediately after the wedge resection, the nodules were imaged with EIS on the back table. Figure 5(a) shows the imaging setup of EIS with a 10 mm endoscope for the back-table imaging of lung cancer nodules in the operating room. We used a 785 nm laser to excite the probes and acquired NIR fluorescent images.

Figure 5(b) shows representative intraoperative visible and NIR fluorescence images of the malignant nodules. Since the photo response in the visible spectrum of the bioinspired image sensor is different than typical color cameras employing Bayer pattern filters, the color rendering of the *ex vivo* specimens are dominated by red color. There is a need for specialized color calibration techniques that can adjust the photo response of the top, middle, and bottom photodiodes to produce responses similar to Bayer pattern filters. These techniques are presently under investigation.

High-intensity fluorescence was detected from tumors during the surgery that correlates well with back table images [Fig. 5(c)], whereas no significant fluorescence was detected from the adjacent normal tissues. EIS was able to differentiate the malignant nodules from the background based on the fluorescence with a 95% tolerance level [Fig. 5(d)], thereby providing high contrast between the tumor and background with a mean TBR of 4.73±0.44. All six tumor regions from the six specimens exhibited significantly higher fluorescence compared to the background. Figure 5(e) displays the hue-saturation response of the fluorescence signal from the nodules which corresponds to the response from ICG in a vial. The H&E [Fig. 5(f)] and fluorescence images [Fig. 5(g)] of the tumor nodule and normal lobe validate that high-intensity fluorescence was associated with the presence of malignant cells.

## 4 Discussion

Surgery remains the primary curative option for patients with solid cancers, but complete removal of the tumor mass and involved lymphatic nodes after early detection remains a major clinical challenge. Despite the latest advancements in tumor-targeted molecular probes and imaging devices, incomplete tumor resection rates are still alarmingly high. One of the major obstacles in surgeries that utilize a single molecular tracer is the inter- and intra-tumor biomarker heterogeneity, which can limit tumor detection rates. For example, nearly 80% of lung adenocarcinomas have high expression of folate alpha receptors and can benefit from the pafolacianine dye to highlight the location of tumors during surgical procedures.^43^^–^^45^ It has been clinically demonstrated that in this patient population, unplanned secondary tumor nodules were identified in ∼50% of the patient, attesting to the benefits of fluorescence-guided surgery (FGS). Although a 5-year disease-free survival rate is still unknown for patients undergoing FGS with pafolacianine, the improved detection rate of secondary tumors provides optimism toward a better clinical outcome. However, about ∼20% of adenocarcinoma patients and the majority of squamous cell and non-small cell lung cancer patients will not benefit from the pafolacianine probe due to a lack of folate alpha receptors in the primary tumors. Enzyme- or pH-activated probes have the potential to address some of these shortcomings as demonstrated by results from phase 2 and phase 3 clinical trials, though none of these probes are FDA-approved at this time.^46^^,^^47^ Since there is still no universal molecular probe that works for all types of cancers, the next frontier for FGS will involve a cocktail of carefully selected tracers to highlight all tumors and involved lymph nodes.

Advancements in imaging devices, such as camera sensor miniaturization, fiber illumination, and optical waveguides among others, have opened new possibilities for minimally invasive surgeries. Current state-of-the-art endoscopic imaging instruments are typically optimized to image single NIR probes in the 800 nm range, which is also known as the ICG-channel. However, the single molecular probe approach comes with its own deficits, including highlighting healthy tissues as tumors (false positives) and missing tumors (false negatives) due to inter- and intra-tumor heterogeneity. Combining multiple tumor-specific tracers can reduce the false positive and false negative detection rates while also increasing the confidence in the true negative and true positive signals by accumulating in the tumors together and avoiding healthy tissue. To improve tumor detection sensitivity and specificity, an imaging instrument should be able to detect and differentiate the fluorescence from multiple molecular probes. Aggregating the fluorescence from different probes without individually differentiating them will dampen the strengths of the individual probes to detect tumors and avoid surrounding healthy tissue.

Our group has taken a radically different approach toward designing color-NIR imaging instruments to address several of these shortcomings. Based on the visual system of the mantis shrimp, we have created a single-chip camera that can capture multiple fluorophores in the NIR spectrum. One of the distinct features of our technology is the capability to differentiate two NIR fluorophores excited with the same laser light excitation. Although the clinical value of such probes is still to be explored, the imaging device must be first validated in the lab and clinical-like settings before any clinical trial can be implemented.

Integrating single-chip multispectral cameras with FDA-approved rigid endoscopes can yield suboptimal results. The design of rigid endoscopes requires careful optimization and balancing tradeoffs between high transmission in the NIR and visible spectrum, minimizing chromatic aberrations, and others. Chromatic aberrations are currently corrected by individually adjusting the focus of the NIR and color cameras in FDA-approved endoscopic systems. However, since this is not an option for single-chip multispectral cameras, one of the imaging modalities will appear out of focus. Typically, color images will appear in focus, whereas NIR images will be blurred, thus leading to reduced spatial resolution and decreased fluorescence sensitivity.

To address the current limitations of multispectral endoscopic systems, we have designed a single-chip, color-NIR EIS and clinically validated it on a back table in the operating room. Our system combined an optimized color-NIR rigid endoscope with multi-illumination fiber and BIS at the proximal end. The multiple optical coating on the individual lenses within the rigid endoscopes ensures that both visible and NIR photons are transmitted with high efficiency and focused on the same imaging plane. Due to this optimization, our imaging system has demonstrated 60% higher spatial resolution compared to an FDA-approved endoscope, which can help detect smaller cancerous lesions. Furthermore, this EIS enables multispectral discrimination of multiple narrow-band NIR targets as well as multiple fluorescent probes.

In this paper, we also present pre-clinical and clinical data acquired with EIS. In a pre-clinical setting, we demonstrate spectral discrimination of two small molecule-based tumor-targeted probes: integrin-specific peptides conjugated with IRDye 680RD and EGFR-binding affibodies conjugated with IRDye 800CW. In both benchtop vial experiments and animal models of breast cancer, we demonstrated that in addition to differentiating the presence of individual probes, we could also determine the ratio of the individual tracers. This can provide invaluable information during surgeries where biomarker concentrations can further improve the confidence of the presence or absence of tumors. One of the major challenges for this imaging technology is the high read-out noise of the imaging device ($∼{70e}^{-}$). which reduces the limit of detection and increases the exposure time of the sensor. Since the full well capacity of the middle photodiode is $∼100\;{\mathrm{ke}}^{-}$, the maximum signal-to-noise ratio for the sensor is ∼50  dB. When imaging signals in low-light conditions (for example, weak fluorescence signals), the high level of readout noise from the sensor imposes a limit on the detection rate. Current state-of-the-art imaging sensors has read out noise approaching ${1e}^{-}$, which is due to the use advanced correlated double sampling circuits and in pixel photon amplification to minimize electronics noise. Since the vertically stacked image sensor does not employ any of these advanced electronics circuits, the read-out noise is high and leads to low signal-to-noise ratio imaging in low light conditions. As the read-out electronics for this imaging technology further improve and incorporate known noise cancellation circuits that are used in modern imagers, real-time frame rates and lower limits of detections will be achieved in clinically relevant illumination scenarios.

Finally, our clinical data on the back table in the operating room enabled us to evaluate our endoscopic system in a clinically relevant setting. Although this clinical study was designed to use a single fluorescent probe, these experiments helped us to validate the simultaneous imaging of both NIR fluorescence and color targets, as well as the efficacy of detecting tumors with tumor-targeted probes. As the molecular imaging field moves toward a multi-tumor targeted probe approach, our imaging instrument can facilitate the evaluation of this idea in the operating room.
